# Supplementary material for: The impact of the Covid-19 pandemic on the incidence of diseases and the provision of primary care: A registry-based study
Source: PLoS One. 2022 Jul 6;17(7):e0271049. doi: 10.1371/journal.pone.0271049 (PMC9258821; doi:10.1371/journal.pone.0271049)
Supplement: S1 Appendix — (DOCX) [file pone.0271049.s001.docx]

Appendix 1: ICPC codes used in the study

ICPC codes are divided into two main groups, acute and longterm-chronic, for the analysis presented in figure 1,2,3 and 4. Eight subgroups were created for the analysis in Table 1.

TODO: Referentie toevoegen naar PDF waaruit we de labels hebben gehaald

| ICPC | Label | Group | Subgroup |
| --- | --- | --- | --- |
| A01 | Pain general/multiple sites | Acute |  |
| A02 | Chills | Acute |  |
| A03 | Fever | Acute |  |
| A04 | Weakness/tiredness general | Acute |  |
| A05 | Feeling ill | Acute |  |
| A06 | Fainting/syncope | Acute |  |
| A07 | Coma | Acute |  |
| A08 | Swelling | Acute |  |
| A09 | Sweating problem | Longterm - Chronic | |
| A10 | Bleeding/haemorrhage NOS | Acute |  |
| A11 | Chest pain NOS | Acute |  |
| A13 | Concern/fear medical treatment | Acute |  |
| A16 | Irritable infant | Acute |  |
| A18 | Concern about appearance | Acute |  |
| A20 | Euthanasia request/discussion | Longterm - Chronic | |
| A21 | Risk factor for malignancy | Longterm - Chronic | |
| A23 | Risk factor NOS | Longterm - Chronic | |
| A25 | Fear of death/dying | Acute |  |
| A26 | Fear of cancer NOS | Acute |  |
| A27 | Fear of other disease NOS | Acute |  |
| A28 | Limited function/disability NOS | Longterm - Chronic | |
| A29 | General symptom/complaint other | Acute |  |
| A70 | Tuberculosis | Longterm - Chronic | |
| A71 | Measles | Acute |  |
| A72 | Chickenpox | Acute |  |
| A73 | Malaria | Acute |  |
| A74 | Rubella | Acute |  |
| A75 | Infectious mononucleosis | Acute |  |
| A76 | Viral exanthem other | Acute |  |
| A77 | Viral disease other/NOS | Acute |  |
| A78 | Infectious disease other/NOS | Acute |  |
| A79 | Malignancy NOS | Longterm - Chronic | Cancer |
| A80 | Trauma/injury NOS | Acute |  |
| A81 | Multiple trauma/injuries | Acute |  |
| A82 | Secondary effect of trauma | Longterm - Chronic | |
| A84 | Poisoning by medical agent | Acute |  |
| A85 | Adverse effect medical agent | Acute |  |
| A86 | Toxic effect non-medicinal substance | Acute |  |
| A87 | Complication of medical treatment | Acute |  |
| A88 | Adverse effect physical factor | Acute |  |
| A89 | Effect prosthetic device | Acute |  |
| A90 | Congenital anomaly OS/multiple | Longterm - Chronic | |
| A91 | Abnormal result investigation NOS | Acute |  |
| A92 | Allergy/allergic reaction NOS | Longterm - Chronic | |
| A93 | Premature newborn | Longterm - Chronic | |
| A94 | Perinatal morbidity other | Acute |  |
| A95 | Perinatal mortality | Acute |  |
| A96 | Death | Acute |  |
| A97 | No disease | Acute |  |
| A98 | Health maintenance/prevention | Acute |  |
| A99 | General disease NOS | Longterm - Chronic | |
| B02 | Lymph gland(s) enlarged/painful | Acute |  |
| B04 | Blood symptom/complaint | Acute |  |
| B25 | Fear of aids/HIV | Acute |  |
| B26 | Fear cancer blood/lymph | Acute |  |
| B27 | Fear blood/lymph disease other | Acute |  |
| B28 | Limited function/disability | Longterm - Chronic | |
| B29 | Sympt/complt lymph/immune other | Acute |  |
| B70 | Lymphadenitis acute | Acute |  |
| B71 | Lymphadenitis non-specific | Acute |  |
| B72 | Hodgkin's disease/lymphoma | Longterm - Chronic | Cancer |
| B73 | Leukaemia | Longterm - Chronic | Cancer |
| B74 | Malignant neoplasm blood other | Longterm - Chronic | Cancer |
| B75 | Benign/unspecified neoplasm blood | Longterm - Chronic | |
| B76 | Ruptured spleen traumatic | Acute |  |
| B77 | Injury blood/lymph/spleen other | Acute |  |
| B78 | Hereditary haemolytic anaemia | Longterm - Chronic | |
| B79 | Congen.anom. blood/lymph other | Longterm - Chronic | |
| B80 | Iron deficiency anaemia | Longterm - Chronic | |
| B81 | Anaemia, Vitamin B12/folate def. | Longterm - Chronic | |
| B82 | Anaemia other/unspecified | Longterm - Chronic | |
| B83 | Purpura/coagulation defect | Longterm - Chronic | |
| B84 | Unexplained abnormal white cells | Acute |  |
| B87 | Splenomegaly | Longterm - Chronic | |
| B90 | HIV-infection/aids | Longterm - Chronic | |
| B99 | Blood/lymph/spleen disease other | Longterm - Chronic | |
| D01 | Abdominal pain/cramps general | Acute |  |
| D02 | Abdominal pain epigastric | Acute |  |
| D03 | Heartburn | Acute |  |
| D04 | Rectal/anal pain | Acute |  |
| D05 | Perianal itching | Acute |  |
| D06 | Abdominal pain localized other | Acute |  |
| D07 | Dyspepsia/indigestion | Acute |  |
| D08 | Flatulence/gas/belching | Acute |  |
| D09 | Nausea | Acute |  |
| D10 | Vomiting | Acute |  |
| D11 | Diarrhoea | Acute |  |
| D12 | Constipation | Longterm - Chronic | |
| D13 | Jaundice | Acute |  |
| D14 | Haematemesis/vomiting blood | Acute |  |
| D15 | Melaena | Acute |  |
| D16 | Rectal bleeding | Acute |  |
| D17 | Incontinence of bowel | Longterm - Chronic | |
| D18 | Change faeces/bowel movements | Acute |  |
| D19 | Teeth/gum symptom/complaint | Acute |  |
| D20 | Mouth/tongue/lip symptom/complt. | Acute |  |
| D21 | Swallowing problem | Acute |  |
| D23 | Hepatomegaly | Longterm - Chronic | |
| D24 | Abdominal mass NOS | Acute |  |
| D25 | Abdominal distension | Acute |  |
| D26 | Fear of cancer of digestive system | Acute |  |
| D27 | Fear of digestive disease other | Acute |  |
| D28 | Limited function/disability (d) | Longterm - Chronic | |
| D29 | Digestive symptom/complaint other | Acute |  |
| D70 | Gastrointestinal infection | Acute |  |
| D71 | Mumps | Acute |  |
| D72 | Viral hepatitis | Longterm - Chronic | Chronic liver |
| D73 | Gastroenteritis presumed infection | Acute |  |
| D74 | Malignant neoplasm stomach | Longterm - Chronic | Cancer |
| D75 | Malignant neoplasm colon/rectum | Longterm - Chronic | Cancer |
| D76 | Malignant neoplasm pancreas | Longterm - Chronic | Cancer |
| D77 | Malig. neoplasm digest other/NOS | Longterm - Chronic | Cancer |
| D78 | Neoplasm digest benign/uncertain | Acute |  |
| D79 | Foreign body digestive system | Acute |  |
| D80 | Injury digestive system other | Acute |  |
| D81 | Congen. anomaly digestive system | Longterm - Chronic | Chronic liver |
| D82 | Teeth/gum disease | Acute |  |
| D83 | Mouth/tongue/lip disease | Acute |  |
| D84 | Oesophagus disease | Longterm - Chronic | |
| D85 | Duodenal ulcer | Acute |  |
| D86 | Peptic ulcer other | Acute |  |
| D87 | Stomach function disorder | Longterm - Chronic | |
| D88 | Appendicitis | Acute |  |
| D89 | Inguinal hernia | Longterm - Chronic | |
| D90 | Hiatus hernia | Longterm - Chronic | |
| D91 | Abdominal hernia other | Longterm - Chronic | |
| D92 | Diverticular disease | Acute |  |
| D93 | Irritable bowel syndrome | Longterm - Chronic | |
| D94 | Chronic enteritis/ulcerative colitis | Longterm - Chronic | |
| D95 | Anal fissure/perianal abscess | Longterm - Chronic | |
| D96 | Worms/other parasites | Acute |  |
| D97 | Liver disease NOS | Longterm - Chronic | Chronic liver |
| D98 | Cholecystitis/cholelithiasis | Longterm - Chronic | |
| D99 | Disease digestive system, other | Longterm - Chronic | |
| F01 | Eye pain | Acute |  |
| F02 | Red eye | Acute |  |
| F03 | Eye discharge | Acute |  |
| F04 | Visual floaters/spots | Acute |  |
| F05 | Visual disturbance other | Acute |  |
| F13 | Eye sensation abnormal | Acute |  |
| F14 | Eye movements abnormal | Acute |  |
| F15 | Eye appearance abnormal | Acute |  |
| F16 | Eyelid symptom/complaint | Acute |  |
| F17 | Glasses symptom/complaint | Acute |  |
| F18 | Contact lens symptom/complaint | Acute |  |
| F27 | Fear of eye disease | Acute |  |
| F28 | Limited function/disability (f) | Longterm - Chronic | |
| F29 | Eye symptom/complaint other | Acute |  |
| F70 | Conjunctivitis infectious | Acute |  |
| F71 | Conjunctivitis allergic | Longterm - Chronic | |
| F72 | Blepharitis/stye/chalazion | Acute |  |
| F73 | Eye infection/inflammation other | Acute |  |
| F74 | Neoplasm of eye/adnexa | Longterm - Chronic | Cancer |
| F75 | Contusion/haemorrhage eye | Acute |  |
| F76 | Foreign body in eye | Acute |  |
| F79 | Injury eye other | Acute |  |
| F80 | Blocked lacrimal duct of infant | Acute |  |
| F81 | Congenital anomaly eye other | Longterm - Chronic | |
| F82 | Detached retina | Longterm - Chronic | |
| F83 | Retinopathy | Longterm - Chronic | |
| F84 | Macular degeneration | Longterm - Chronic | |
| F85 | Corneal ulcer | Acute |  |
| F86 | Trachoma | Acute |  |
| F91 | Refractive error | Longterm - Chronic | |
| F92 | Cataract | Longterm - Chronic | |
| F93 | Glaucoma | Longterm - Chronic | |
| F94 | Blindness | Longterm - Chronic | |
| F95 | Strabismus | Longterm - Chronic | |
| F99 | Eye/adnexa disease, other | Longterm - Chronic | |
| H01 | Ear pain/earache | Acute |  |
| H02 | Hearing complaint | Acute |  |
| H03 | Tinnitus, ringing/buzzing ear | Longterm - Chronic | |
| H04 | Ear discharge | Acute |  |
| H05 | Bleeding ear | Acute |  |
| H13 | Plugged feeling ear | Acute |  |
| H15 | Concern with appearance of ears | Acute |  |
| H27 | Fear of ear disease | Acute |  |
| H28 | Limited function/disability ear | Longterm - Chronic | |
| H29 | Ear symptom/complaint other | Acute |  |
| H70 | Otitis externa | Acute |  |
| H71 | Acute otitis media/myringitis | Acute |  |
| H72 | Serous otitis media | Acute |  |
| H73 | Eustachian salpingitis | Acute |  |
| H74 | Chronic otitis media | Longterm - Chronic | |
| H75 | Neoplasm of ear | Longterm - Chronic | Cancer |
| H76 | Foreign body in ear | Acute |  |
| H77 | Perforation ear drum | Longterm - Chronic | |
| H78 | Superficial injury of ear | Acute |  |
| H79 | Ear injury other | Acute |  |
| H80 | Congenital anomaly of ear | Longterm - Chronic | |
| H81 | Excessive ear wax | Acute |  |
| H82 | Vertiginous syndrome | Longterm - Chronic | |
| H83 | Otosclerosis | Longterm - Chronic | |
| H84 | Presbyacusis | Longterm - Chronic | |
| H85 | Acoustic trauma | Longterm - Chronic | |
| H86 | Deafness | Longterm - Chronic | |
| H99 | Ear/mastoid disease, other | Longterm - Chronic | |
| K01 | Heart pain | Acute |  |
| K02 | Pressure/tightness of heart | Acute |  |
| K03 | Cardiovascular pain NOS | Acute |  |
| K04 | Palpitations/awareness of heart | Acute |  |
| K05 | Irregular heartbeat other | Acute |  |
| K06 | Prominent veins | Longterm - Chronic | |
| K07 | Swollen ankles/oedema | Acute |  |
| K22 | Risk factor cardiovascular disease | Longterm - Chronic | |
| K24 | Fear of heart disease | Acute |  |
| K25 | Fear of hypertension | Acute |  |
| K27 | Fear cardiovascular disease other | Acute |  |
| K28 | Limited function/disability (k) | Longterm - Chronic | |
| K29 | Cardiovascular sympt./complt. other | Acute |  |
| K70 | Infection of circulatory system | Longterm - Chronic | |
| K71 | Rheumatic fever/heart disease | Longterm - Chronic | |
| K72 | Neoplasm cardiovascular | Longterm - Chronic | Cancer |
| K73 | Congenital anomaly cardiovascular | Longterm - Chronic | Chronic caridovascular |
| K74 | Ischaemic heart disease w. angina | Longterm - Chronic | Chronic caridovascular |
| K75 | Acute myocardial infarction | Longterm - Chronic | Chronic caridovascular |
| K76 | Ischaemic heart disease w/o angina | Longterm - Chronic | Chronic caridovascular |
| K77 | Heart failure | Longterm - Chronic | Chronic caridovascular |
| K78 | Atrial fibrillation/flutter | Longterm - Chronic | Chronic caridovascular |
| K79 | Paroxysmal tachycardia | Longterm - Chronic | |
| K80 | Cardiac arrhythmia NOS | Longterm - Chronic | |
| K81 | Heart/arterial murmur NOS | Longterm - Chronic | |
| K82 | Pulmonary heart disease | Longterm - Chronic | Chronic caridovascular |
| K83 | Heart valve disease NOS | Longterm - Chronic | Chronic caridovascular |
| K84 | Heart disease other | Longterm - Chronic | |
| K85 | Elevated blood pressure | Acute |  |
| K86 | Hypertension uncomplicated | Longterm - Chronic | Chronic caridovascular |
| K87 | Hypertension complicated | Longterm - Chronic | Chronic caridovascular |
| K88 | Postural hypotension | Longterm - Chronic | |
| K89 | Transient cerebral ischaemia | Longterm - Chronic | |
| K90 | Stroke/cerebrovascular accident | Longterm - Chronic | Chronic caridovascular |
| K91 | Cerebrovascular disease | Longterm - Chronic | Chronic caridovascular |
| K92 | Atherosclerosis/PVD | Longterm - Chronic | Chronic caridovascular |
| K93 | Pulmonary embolism | Longterm - Chronic | Chronic caridovascular |
| K94 | Phlebitis/thrombophlebitis | Longterm - Chronic | |
| K95 | Varicose veins of leg | Longterm - Chronic | |
| K96 | Haemorrhoids | Longterm - Chronic | |
| K99 | Cardiovascular disease other | Longterm - Chronic | |
| L01 | Neck symptom/complain | Acute |  |
| L02 | Back symptom/complaint | Acute |  |
| L03 | Low back symptom/complaint | Acute |  |
| L04 | Chest symptom/complaint | Acute |  |
| L05 | Flank/axilla symptom/complaint | Acute |  |
| L07 | Jaw symptom/complaint | Acute |  |
| L08 | Shoulder symptom/complaint | Acute |  |
| L09 | Arm symptom/complaint | Acute |  |
| L10 | Elbow symptom/complaint | Acute |  |
| L11 | Wrist symptom/complaint | Acute |  |
| L12 | Hand/finger symptom/complaint | Acute |  |
| L13 | Hip symptom/complaint | Acute |  |
| L14 | Leg/thigh symptom/complaint | Acute |  |
| L15 | Knee symptom/complaint | Acute |  |
| L16 | Ankle symptom/complaint | Acute |  |
| L17 | Foot/toe symptom/complaint | Acute |  |
| L18 | Muscle pain | Acute |  |
| L19 | Muscle symptom/complaint NOS | Acute |  |
| L20 | Joint symptom/complaint NOS | Acute |  |
| L26 | Fear of cancer musculoskeletal | Acute |  |
| L27 | Fear musculoskeletal disease other | Acute |  |
| L28 | Limited function/disability (l) | Longterm - Chronic | |
| L29 | Sympt/complt. Musculoskeletal other | Acute |  |
| L70 | Infections musculoskeletal system | Acute |  |
| L71 | Malignant neoplasm musculoskeletal | Longterm - Chronic | Cancer |
| L72 | Fracture: radius/ulna | Acute |  |
| L73 | Fracture: tibia/fibula | Longterm - Chronic | |
| L74 | Fracture: hand/foot bone | Acute |  |
| L75 | Fracture: femur | Longterm - Chronic | |
| L76 | Fracture: other | Longterm - Chronic | |
| L77 | Sprain/strain of ankle | Acute |  |
| L78 | Sprain/strain of knee | Acute |  |
| L79 | Sprain/strain of joint NOS | Acute |  |
| L80 | Dislocation/subluxation | Acute |  |
| L81 | Injury musculoskeletal NOS | Acute |  |
| L82 | Congenital anomaly musculoskeletal | Longterm - Chronic | |
| L83 | Neck syndrome | Longterm - Chronic | |
| L84 | Back syndrome w/o radiating pain | Longterm - Chronic | |
| L85 | Acquired deformity of spine | Longterm - Chronic | |
| L86 | Back syndrome with radiating pain | Longterm - Chronic | |
| L87 | Bursitis/tendinitis/synovitis NOS | Acute |  |
| L88 | Rheumatoid/seropositive arthritis | Longterm - Chronic | |
| L89 | Osteoarthrosis of hip | Longterm - Chronic | |
| L90 | Osteoarthrosis of knee | Longterm - Chronic | |
| L91 | Osteoarthrosis other | Longterm - Chronic | |
| L92 | Shoulder syndrome | Longterm - Chronic | |
| L93 | Tennis elbow | Longterm - Chronic | |
| L94 | Osteochondrosis | Longterm - Chronic | |
| L95 | Osteoporosis | Longterm - Chronic | |
| L96 | Acute internal damage knee | Acute |  |
| L97 | Neoplasm benign/unspec musculo. | Acute |  |
| L98 | Acquired deformity of limb | Longterm - Chronic | |
| L99 | Musculoskeletal disease, other | Longterm - Chronic | |
| N01 | Headache | Acute |  |
| N03 | Pain face | Acute |  |
| N04 | Restless legs | Longterm - Chronic | |
| N05 | Tingling fingers/feet/toes | Acute |  |
| N06 | Sensation disturbance other | Acute |  |
| N07 | Convulsion/seizure | Acute |  |
| N08 | Abnormal involuntary movements | Acute |  |
| N16 | Disturbance of smell/taste | Acute |  |
| N17 | Vertigo/dizziness | Acute |  |
| N18 | Paralysis/weakness | Longterm - Chronic | |
| N19 | Speech disorder | Longterm - Chronic | |
| N26 | Fear cancer neurological system | Acute |  |
| N27 | Fear of neurological disease other | Acute |  |
| N28 | Limited function/disability (n) | Longterm - Chronic | Chronic neurological |
| N29 | Neurological symptom/complt. other | Acute |  |
| N70 | Poliomyelitis | Longterm - Chronic | |
| N71 | Meningitis/encephalitis | Longterm - Chronic | |
| N72 | Tetanus | Acute |  |
| N73 | Neurological infection other | Longterm - Chronic | |
| N74 | Malignant neoplasm nervous system | Longterm - Chronic | Cancer |
| N75 | Benign neoplasm nervous system | Acute |  |
| N76 | Neoplasm nervous system unspec. | Acute | Cancer |
| N79 | Concussion | Acute |  |
| N80 | Head injury other | Acute |  |
| N81 | Injury nervous system other | Acute |  |
| N85 | Congenital anomaly neurological | Longterm - Chronic | |
| N86 | Multiple sclerosis | Longterm - Chronic | Chronic neurological |
| N87 | Parkinsonism | Longterm - Chronic | Chronic neurological |
| N88 | Epilepsy | Longterm - Chronic | Chronic neurological |
| N89 | Migraine | Longterm - Chronic | |
| N90 | Cluster headache | Longterm - Chronic | |
| N91 | Facial paralysis/bell's palsy | Longterm - Chronic | |
| N92 | Trigeminal neuralgia | Longterm - Chronic | |
| N93 | Carpal tunnel syndrome | Longterm - Chronic | |
| N94 | Peripheral neuritis/neuropathy | Longterm - Chronic | |
| N95 | Tension headache | Acute |  |
| N99 | Neurological disease, other | Longterm - Chronic | Chronic neurological |
| P01 | Feeling anxious/nervous/tense | Acute |  |
| P02 | Acute stress reaction | Acute |  |
| P03 | Feeling depressed | Acute |  |
| P04 | Feeling/behaving irritable/angry | Acute |  |
| P05 | Senility, feeling/behaving old | Acute |  |
| P06 | Sleep disturbance | Acute |  |
| P07 | Sexual desire reduced | Acute |  |
| P08 | Sexual fulfilment reduced | Acute |  |
| P09 | Sexual preference concern | Acute |  |
| P10 | Stammering/stuttering/tic | Longterm - Chronic | |
| P11 | Eating problem in child | Longterm - Chronic | |
| P12 | Bedwetting/enuresis | Longterm - Chronic | |
| P13 | Encopresis/bowel training problem | Longterm - Chronic | |
| P15 | Chronic alcohol abuse | Longterm - Chronic | |
| P16 | Acute alcohol abuse | Longterm - Chronic | |
| P17 | Tobacco abuse | Longterm - Chronic | |
| P18 | Medication abuse | Longterm - Chronic | |
| P19 | Drug abuse | Longterm - Chronic | |
| P20 | Memory disturbance | Longterm - Chronic | |
| P22 | Child behaviour symptom/complaint | Longterm - Chronic | |
| P23 | Adolescent behav. Symptom/complt. | Longterm - Chronic | |
| P24 | Specific learning problem | Longterm - Chronic | |
| P25 | Phase of life problem adult | Longterm - Chronic | |
| P27 | Fear of mental disorder | Acute |  |
| P28 | Limited function/disability (p) | Longterm - Chronic | |
| P29 | Psychological symptom/complt other | Acute |  |
| P70 | Dementia | Longterm - Chronic | Chronic neurological |
| P71 | Organic psychosis other | Longterm - Chronic | |
| P72 | Schizophrenia | Longterm - Chronic | |
| P73 | Affective psychosis | Longterm - Chronic | |
| P74 | Anxiety disorder/anxiety state | Longterm - Chronic | |
| P75 | Somatization disorder | Longterm - Chronic | |
| P76 | Depressive disorder | Longterm - Chronic | |
| P77 | Suicide/suicide attempt | Acute |  |
| P78 | Neuraesthenia/surmenage | Longterm - Chronic | |
| P79 | Phobia/compulsive disorder | Longterm - Chronic | |
| P80 | Personality disorder | Longterm - Chronic | |
| P81 | Hyperkinetic disorder | Longterm - Chronic | |
| P82 | Post-traumatic stress disorder | Longterm - Chronic | |
| P85 | Mental retardation | Longterm - Chronic | |
| P86 | Anorexia nervosa/bulimia | Longterm - Chronic | |
| P98 | Psychosis NOS/other | Longterm - Chronic | |
| P99 | Psychological disorders, other | Longterm - Chronic | |
| R01 | Pain respiratory system | Acute |  |
| R02 | Shortness of breath/dyspnoea | Acute |  |
| R03 | Wheezing | Acute |  |
| R04 | Breathing problem, other | Acute |  |
| R05 | Cough | Acute |  |
| R06 | Nose bleed/epistaxis | Acute |  |
| R07 | Sneezing/nasal congestion | Acute |  |
| R08 | Nose symptom/complaint other | Acute |  |
| R09 | Sinus symptom/complaint | Acute |  |
| R21 | Throat symptom/complaint | Acute |  |
| R23 | Voice symptom/complaint | Acute |  |
| R24 | Haemoptysis | Acute |  |
| R25 | Sputum/phlegm abnormal | Acute |  |
| R26 | Fear of cancer respiratory system | Acute |  |
| R27 | Fear of respiratory disease, other | Acute |  |
| R28 | Limited function/disability (r) | Longterm - Chronic | Chronic lung |
| R29 | Respiratory symptom/complaint oth. | Acute |  |
| R71 | Whooping cough | Acute |  |
| R72 | Strep throat | Acute |  |
| R73 | Boil/abscess nose | Acute |  |
| R74 | Upper respiratory infection acute | Acute |  |
| R75 | Sinusitis acute/chronic | Acute |  |
| R76 | Tonsillitis acute | Acute |  |
| R77 | Laryngitis/tracheitis acute | Acute |  |
| R78 | Acute bronchitis/bronchiolitis | Acute |  |
| R79 | Chronic bronchitis | Longterm - Chronic | Chronic lung |
| R80 | Influenza | Acute |  |
| R81 | Pneumonia | Acute |  |
| R82 | Pleurisy/pleural effusion | Acute |  |
| R83 | Respiratory infection other | Acute |  |
| R84 | Malignant neoplasm bronchus/lung | Longterm - Chronic | Cancer |
| R85 | Malinant neoplasm respiratory, other | Longterm - Chronic | Cancer |
| R86 | Benign neoplasm respiratory | Acute |  |
| R87 | Foreign body nose/larynx/bronch | Acute |  |
| R88 | Injury respiratory other | Acute |  |
| R89 | Congenital anomaly respiratory | Longterm - Chronic | |
| R90 | Hypertrophy tonsils/adenoids | Longterm - Chronic | |
| R92 | Neoplasm respiratory unspecified | Longterm - Chronic | Cancer |
| R95 | Chronic obstructive pulmonary dis | Longterm - Chronic | Chronic lung |
| R96 | Asthma | Longterm - Chronic | Chronic lung |
| R97 | Allergic rhinitis | Longterm - Chronic | |
| R98 | Hyperventilation syndrome | Longterm - Chronic | |
| R99 | Respiratory disease other | Longterm - Chronic | |
| S01 | Pain/tenderness of skin | Acute |  |
| S02 | Pruritus | Acute |  |
| S03 | Warts | Acute |  |
| S04 | Lump/swelling localized | Acute |  |
| S05 | Lumps/swellings generalized | Acute |  |
| S06 | Rash localized | Acute |  |
| S07 | Rash generalized | Acute |  |
| S08 | Skin colour change | Acute |  |
| S09 | Infected finger/toe | Acute |  |
| S10 | Boil/carbuncle | Acute |  |
| S11 | Skin infection post-traumatic | Acute |  |
| S12 | Insect bite/sting | Acute |  |
| S13 | Animal/human bite | Acute |  |
| S14 | Burn/scald | Acute |  |
| S15 | Foreign body in skin | Acute |  |
| S16 | Bruise/contusion | Acute |  |
| S17 | Abrasion/scratch/blister | Acute |  |
| S18 | Laceration/cut | Acute |  |
| S19 | Skin injury other | Acute |  |
| S20 | Corn/callosity | Acute |  |
| S21 | Skin texture symptom/complaint | Acute |  |
| S22 | Nail symptom/complaint | Acute |  |
| S23 | Hair loss/baldness | Acute |  |
| S24 | Hair/scalp symptom/complaint | Acute |  |
| S26 | Fear of cancer of skin | Acute |  |
| S27 | Fear of skin disease other | Acute |  |
| S28 | Limited function/disability (s) | Longterm - Chronic | |
| S29 | Skin symptom/complaint other | Acute |  |
| S70 | Herpes zoster | Acute |  |
| S71 | Herpes simplex | Acute |  |
| S72 | Scabies/other acariasis | Acute |  |
| S73 | Pediculosis/skin infestation other | Acute |  |
| S74 | Dermatophytosis | Acute |  |
| S75 | Moniliasis/candidiasis skin | Acute |  |
| S76 | Skin infection other | Acute |  |
| S77 | Malignant neoplasm of skin | Longterm - Chronic | Cancer |
| S78 | Lipoma | Acute |  |
| S79 | Neoplasm skin benign/unspecified | Longterm - Chronic | |
| S80 | Solar keratosis/sunburn | Acute |  |
| S81 | Haemangioma/lymphangioma | Longterm - Chronic | |
| S82 | Naevus/mole | Longterm - Chronic | |
| S83 | Congenital skin anomaly other | Longterm - Chronic | |
| S84 | Impetigo | Acute |  |
| S85 | Pilonidal cyst/fistula | Acute |  |
| S86 | Dermatitis seborrhoeic | Longterm - Chronic | |
| S87 | Dermatitis/atopic eczema | Longterm - Chronic | |
| S88 | Dermatitis contact/allergic | Longterm - Chronic | |
| S89 | Diaper rash | Acute |  |
| S90 | Pityriasis rosea | Acute |  |
| S91 | Psoriasis | Longterm - Chronic | |
| S92 | Sweat gland disease | Longterm - Chronic | |
| S93 | Sebaceous cyst | Acute |  |
| S94 | Ingrowing nail | Acute |  |
| S95 | Molluscum contagiosum | Longterm - Chronic | |
| S96 | Acne | Longterm - Chronic | |
| S97 | Chronic ulcer skin | Longterm - Chronic | |
| S98 | Urticaria | Longterm - Chronic | |
| S99 | Skin disease, other | Longterm - Chronic | |
| T01 | Excessive thirst | Acute |  |
| T02 | Excessive appetite | Acute |  |
| T03 | Loss of appetite | Acute |  |
| T04 | Feeding problem of infant/child | Acute |  |
| T05 | Feeding problem of adult | Acute |  |
| T07 | Weight gain | Acute |  |
| T08 | Weight loss | Acute |  |
| T10 | Growth delay | Longterm - Chronic | |
| T11 | Dehydration | Acute |  |
| T26 | Fear of cancer of endocrine system | Acute |  |
| T27 | Fear endocrine/metabolic dis other | Acute |  |
| T28 | Limited function/disability (t) | Longterm - Chronic | |
| T29 | Endocrine/met./sympt/complt other | Acute |  |
| T70 | Endocrine infection | Acute |  |
| T71 | Malignant neoplasm thyroid | Longterm - Chronic | Cancer |
| T72 | Benign neoplasm thyroid | Acute |  |
| T73 | Neoplasm endocrine oth/unspecified | Acute | Cancer |
| T78 | Thyroglossal duct/cyst | Longterm - Chronic | |
| T80 | Congenital anom endocrine/metab. | Longterm - Chronic | |
| T81 | Goitre | Longterm - Chronic | |
| T82 | Obesity | Longterm - Chronic | Obesity |
| T83 | Overweight | Longterm - Chronic | |
| T85 | Hyperthyroidism/thyrotoxicosis | Longterm - Chronic | |
| T86 | Hypothyroidism/myxoedema | Longterm - Chronic | |
| T87 | Hypoglycaemia | Acute |  |
| T89 | Diabetes insulin dependent | Longterm - Chronic | Diabetes |
| T90 | Diabetes non-insulin dependent | Longterm - Chronic | Diabetes |
| T91 | Vitamin/nutritional deficiency | Longterm - Chronic | |
| T92 | Gout | Longterm - Chronic | |
| T93 | Lipid disorder | Longterm - Chronic | |
| T99 | Endocrine/metab/nutrit. dis. other | Longterm - Chronic | |
| U01 | Dysuria/painful urination | Acute |  |
| U02 | Urinary frequency/urgency | Acute |  |
| U04 | Incontinence urine | Longterm - Chronic | |
| U05 | Urination problems other | Acute |  |
| U06 | Haematuria | Acute |  |
| U07 | Urine symptom/complaint other | Acute |  |
| U08 | Urinary retention | Acute |  |
| U13 | Bladder symptom/complaint other | Acute |  |
| U14 | Kidney symptom/complaint | Acute |  |
| U26 | Fear of cancer of urinary system | Acute |  |
| U27 | Fear of urinary disease other | Acute |  |
| U28 | Limited function/disability urinary | Longterm - Chronic | Chronic renal |
| U29 | Urinary symptom/complaint other | Acute |  |
| U70 | Pyelonephritis/pyelitis | Acute |  |
| U71 | Cystitis/urinary infection other | Acute |  |
| U72 | Urethritis | Acute |  |
| U75 | Malignant neoplasm of kidney | Longterm - Chronic | Cancer |
| U76 | Malignant neoplasm of bladder | Longterm - Chronic | Cancer |
| U77 | Malignant neoplasm urinary other | Longterm - Chronic | Cancer |
| U78 | Benign neoplasm urinary tract | Acute |  |
| U79 | Neoplasm urinary tract NOS | Longterm - Chronic | Cancer |
| U80 | Injury urinary tract | Acute |  |
| U85 | Congenital anomaly urinary tract | Longterm - Chronic | |
| U88 | Glomerulonephritis/nephrosis | Longterm - Chronic | Chronic renal |
| U90 | Orthostatic albumin./proteinuria | Longterm - Chronic | |
| U95 | Urinary calculus | Longterm - Chronic | |
| U98 | Abnormal urine test NOS | Acute |  |
| U99 | Urinary disease, other | Longterm - Chronic | |
| W01 | Question of pregnancy | Acute |  |
| W02 | Fear of pregnancy | Acute |  |
| W03 | Antepartum bleeding | Acute |  |
| W05 | Pregnancy vomiting/nausea | Acute |  |
| W10 | Contraception postcoital | Acute |  |
| W11 | Contraception oral | Longterm - Chronic | |
| W12 | Contraception intrauterine | Longterm - Chronic | |
| W13 | Sterilization | Longterm - Chronic | |
| W14 | Contraception other | Longterm - Chronic | |
| W15 | Infertility/subfertility | Longterm - Chronic | |
| W17 | Post-partum bleeding | Acute |  |
| W18 | Post-partum symptom/complaint oth. | Acute |  |
| W19 | Breast/lactation symptom/complaint | Acute |  |
| W21 | Concern body image in pregnancy | Acute |  |
| W27 | Fear complications of pregnancy | Acute |  |
| W28 | Limited function/disability (w) | Longterm - Chronic | |
| W29 | Pregnancy symptom/complaint other | Acute |  |
| W70 | Puerperal infection/sepsis | Acute |  |
| W71 | Infection complicating pregnancy | Acute |  |
| W72 | Malignant neoplasm relate to preg. | Longterm - Chronic | Cancer |
| W73 | Benign/unspec. neoplasm/pregnancy | Acute |  |
| W75 | Injury complicating pregnancy | Acute |  |
| W76 | Congenital anomaly complicate preg. | Longterm - Chronic | |
| W78 | Pregnancy | Longterm - Chronic | |
| W79 | Unwanted pregnancy | Longterm - Chronic | |
| W80 | Ectopic pregnancy | Longterm - Chronic | |
| W81 | Toxaemia of pregnancy | Longterm - Chronic | |
| W82 | Abortion spontaneous | Acute |  |
| W83 | Abortion induced | Acute |  |
| W84 | Pregnancy high risk | Longterm - Chronic | |
| W85 | Gestational diabetes | Longterm - Chronic | |
| W90 | Uncomplicate labour/delivery live | Acute |  |
| W91 | Uncomplicate labour/delivery still | Acute |  |
| W92 | Complicate labour/ delivery livebirth | Acute |  |
| W93 | Complicate labour/delivery stillbirth | Acute |  |
| W94 | Puerperal mastitis | Acute |  |
| W95 | Breast disorder in pregnancy other | Acute |  |
| W96 | Complications of puerperium other | Acute |  |
| W99 | Disorder pregnancy/delivery, other | Longterm - Chronic | |
| X01 | Genital pain female | Acute |  |
| X02 | Menstrual pain | Longterm - Chronic | |
| X03 | Intermenstrual pain | Longterm - Chronic | |
| X04 | Painful intercourse female | Longterm - Chronic | |
| X05 | Menstruation absent/scanty | Longterm - Chronic | |
| X06 | Menstruation excessive | Longterm - Chronic | |
| X07 | Menstruation irregular/frequent | Longterm - Chronic | |
| X08 | Intermenstrual bleeding | Longterm - Chronic | |
| X09 | Premenstrual symptom/complaint | Longterm - Chronic | |
| X10 | Postponement of menstruation | Acute |  |
| X11 | Menopausal symptom/complaint | Longterm - Chronic | |
| X12 | Postmenopausal bleeding | Longterm - Chronic | |
| X13 | Postcoital bleeding | Longterm - Chronic | |
| X14 | Vaginal discharge | Acute |  |
| X15 | Vaginal symptom/complaint other | Acute |  |
| X16 | Vulval symptom/complaint | Acute |  |
| X17 | Pelvis symptom/complaint female | Acute |  |
| X18 | Breast pain female | Acute |  |
| X19 | Breast lump/mass female | Acute |  |
| X20 | Nipple symptom/complaint female | Acute |  |
| X21 | Breast symptom/complt. female other | Acute |  |
| X22 | Concern breast appearance female | Acute |  |
| X23 | Fear sexually transmitted disease (f) | Acute |  |
| X24 | Fear of sexual dysfunction female | Acute |  |
| X25 | Fear of genital cancer female | Acute |  |
| X26 | Fear of breast cancer female | Acute |  |
| X27 | Fear genital/breast disease other (f) | Acute |  |
| X28 | Limited function/disability (x) | Longterm - Chronic | |
| X29 | Genital symptom/complt female oth. | Acute |  |
| X70 | Syphilis female | Acute |  |
| X71 | Gonorrhoea female | Acute |  |
| X72 | Genital candidiasis female | Acute |  |
| X73 | Genital trichomoniasis female | Acute |  |
| X74 | Pelvic inflammatory disease | Acute |  |
| X75 | Malignant neoplasm cervix | Longterm - Chronic | Cancer |
| X76 | Malignant neoplasm breast female | Longterm - Chronic | Cancer |
| X77 | Malignant neoplasm genital other (f) | Longterm - Chronic | Cancer |
| X78 | Fibromyoma uterus | Longterm - Chronic | |
| X79 | Benign neoplasm breast female | Longterm - Chronic | |
| X80 | Benign neoplasm female genital | Longterm - Chronic | |
| X81 | Genital neoplasm oth/unspecied (f) | Longterm - Chronic | Cancer |
| X82 | Injury genital female | Acute |  |
| X83 | Congenital anomaly genital female | Longterm - Chronic | |
| X84 | Vaginitis/vulvitis NOS | Acute |  |
| X85 | Cervical disease NOS | Acute |  |
| X86 | Abnormal cervix smear | Longterm - Chronic | |
| X87 | Uterovaginal prolapse | Longterm - Chronic | |
| X88 | Fibrocystic disease breast | Acute |  |
| X89 | Premenstrual tension syndrome | Longterm - Chronic | |
| X90 | Genital herpes female | Acute |  |
| X91 | Condylomata acuminata female | Acute |  |
| X92 | Chlamydia infection genital (f) | Acute |  |
| X99 | Genital disease female, other | Longterm - Chronic | |
| Y01 | Pain in penis | Acute |  |
| Y02 | Pain in testis/scrotum | Acute |  |
| Y03 | Urethral discharge | Acute |  |
| Y04 | Penis symptom/complaint other | Acute |  |
| Y05 | Scrotum/testis sympt/complt. other | Acute |  |
| Y06 | Prostate symptom/complaint | Acute |  |
| Y07 | Impotence NOS | Longterm - Chronic | |
| Y08 | Sexual function sympt./complt.(m) | Acute |  |
| Y10 | Infertility/subfertility male | Longterm - Chronic | |
| Y13 | Sterilization male | Longterm - Chronic | |
| Y14 | Family planning male other | Longterm - Chronic | |
| Y16 | Breast symptom/complaint male | Acute |  |
| Y24 | Fear of sexual dysfunction male | Acute |  |
| Y25 | Fear sexually transmitted dis. male | Acute |  |
| Y26 | Fear of genital cancer male | Acute |  |
| Y27 | Fear of genital disease male other | Acute |  |
| Y28 | Limited function/disability (y) | Longterm - Chronic | |
| Y29 | Genital sympt./complt.male other | Acute |  |
| Y70 | Syphilis male | Acute |  |
| Y71 | Gonorrhoea male | Acute |  |
| Y72 | Genital herpes male | Acute |  |
| Y73 | Prostatitis/seminal vesiculitis | Acute |  |
| Y74 | Orchitis/epididymitis | Acute |  |
| Y75 | Balanitis | Acute |  |
| Y76 | Condylomata acuminata male | Acute |  |
| Y77 | Malignant neoplasm prostate | Longterm - Chronic | Cancer |
| Y78 | Malign neoplasm male genital other | Longterm - Chronic | Cancer |
| Y79 | Benign/unspec. neoplasm gen. (m) | Longterm - Chronic | |
| Y80 | Injury male genital | Acute |  |
| Y81 | Phimosis/redundant prepuce | Longterm - Chronic | |
| Y82 | Hypospadias | Longterm - Chronic | |
| Y83 | Undescended testicle | Longterm - Chronic | |
| Y84 | Congenital genl anomaly (m) other | Longterm - Chronic | |
| Y85 | Benign prostatic hypertrophy | Longterm - Chronic | |
| Y86 | Hydrocoele | Longterm - Chronic | |
| Y99 | Genital disease male, other | Longterm - Chronic | |
| Z01 | Poverty/financial problem | Longterm - Chronic | |
| Z02 | Food/water problem | Longterm - Chronic | |
| Z03 | Housing/neighbourhood problem | Longterm - Chronic | |
| Z04 | Social cultural problem | Longterm - Chronic | |
| Z05 | Work problem | Longterm - Chronic | |
| Z06 | Unemployment problem | Longterm - Chronic | |
| Z07 | Education problem | Longterm - Chronic | |
| Z08 | Social welfare problem | Longterm - Chronic | |
| Z09 | Legal problem | Longterm - Chronic | |
| Z10 | Health care system problem | Longterm - Chronic | |
| Z11 | Compliance/being ill problem | Longterm - Chronic | |
| Z12 | Relationship problem with partner | Longterm - Chronic | |
| Z13 | Partner's behaviour problem | Longterm - Chronic | |
| Z14 | Partner illness problem | Longterm - Chronic | |
| Z15 | Loss/death of partner problem | Longterm - Chronic | |
| Z16 | Relationship problem with child | Longterm - Chronic | |
| Z18 | Illness problem with child | Longterm - Chronic | |
| Z19 | Loss/death of child problem | Longterm - Chronic | |
| Z20 | Relationship prob. parent/family | Longterm - Chronic | |
| Z21 | Behaviour problem parent/family | Longterm - Chronic | |
| Z22 | Illness problem parent/family | Longterm - Chronic | |
| Z23 | Loss/death parent/family member | Longterm - Chronic | |
| Z24 | Relationship problem friend | Longterm - Chronic | |
| Z25 | Assault/harmful event problem | Longterm - Chronic | |
| Z27 | Fear of a social problem | Acute |  |
| Z28 | Limited function/disability (z) | Longterm - Chronic | |
| Z29 | Social problem NOS | Longterm - Chronic | |
