# Supplementary material for: The impact of the Covid-19 pandemic on the incidence of diseases and the provision of primary care: A registry-based study
Source: PLoS One. 2022 Jul 6;17(7):e0271049. doi: 10.1371/journal.pone.0271049 (PMC9258821; doi:10.1371/journal.pone.0271049)
Supplement: S2 Appendix — (DOCX) [file pone.0271049.s002.docx]

Appendix 2 Regression coefficients for the effect of demographic variables on the number of registered health outcomes

2.1 Care intensity

| variable | period | level | reference | estimate | se | 95% CI | Pvalue |
| --- | --- | --- | --- | --- | --- | --- | --- |
| Intercept | 2018-2019 | - | - | 1.72 | 0.01 | 1.70;1.74 | 0 |
| Intercept | 2020 | - | - | 1.81 | 0.01 | 1.79;1.83 | 0 |
| Nationality | 2018-2019 | Foreign | Belgian | -0.08 | 0 | -0.08;-0.07 | 0 |
| Nationality | 2020 | Foreign | Belgian | -0.06 | 0 | -0.07;-0.06 | 0 |
| Sex | 2018-2019 | Male | Female | -0.15 | 0 | -0.15;-0.15 | 0 |
| Sex | 2020 | Male | Female | -0.16 | 0 | -0.16;-0.15 | 0 |
| Age | 2018-2019 | 0-17 | 18-34 | -0.26 | 0 | -0.27;-0.26 | 0 |
| Age | 2018-2019 | 35-65 | 18-34 | 0.22 | 0 | 0.22;0.22 | 0 |
| Age | 2018-2019 | 65+ | 18-34 | 0.57 | 0 | 0.57;0.57 | 0 |
| Age | 2020 | 0-17 | 18-34 | -0.37 | 0 | -0.38;-0.37 | 0 |
| Age | 2020 | 35-65 | 18-34 | 0.22 | 0 | 0.21;0.22 | 0 |
| Age | 2020 | 65+ | 18-34 | 0.54 | 0 | 0.54;0.55 | 0 |
| Socio-economic status | 2018-2019 | Low | High | 0.33 | 0 | 0.32;0.33 | 0 |
| Socio-economic status | 2020 | Low | High | 0.3 | 0 | 0.30;0.31 | 0 |
| Center city | 2018-2019 | Yes | No | 0.01 | 0 | 0.00;0.01 | 0.0062 |
| Center city | 2020 | Yes | No | 0.02 | 0 | 0.02;0.03 | 0 |
| Practice | - | B | A | -0.1 | 0.01 | -0.13;-0.08 | 0 |
| Practice | - | C | A | -0.08 | 0.01 | -0.10;-0.06 | 0 |
| Practice | - | D | A | -0.48 | 0.01 | -0.51;-0.46 | 0 |
| Practice | - | E | A | -0.25 | 0.01 | -0.28;-0.23 | 0 |
| Practice | - | F | A | -0.12 | 0.01 | -0.14;-0.10 | 0 |
| Practice | - | G | A | -0.3 | 0.01 | -0.33;-0.28 | 0 |
| Practice | - | H | A | 0 | 0.01 | -0.03;0.02 | 0.72 |
| Practice | - | I | A | -0.02 | 0.01 | -0.04;0.01 | 0.1333 |
| Practice | - | J | A | 0.02 | 0.01 | -0.00;0.04 | 0.0576 |
| Practice | - | K | A | 0.03 | 0.01 | 0.01;0.05 | 0.0027 |
| Practice | - | L | A | -0.15 | 0.01 | -0.17;-0.13 | 0 |
| Practice | - | M | A | 0.14 | 0.01 | 0.11;0.16 | 0 |
| Practice | - | N | A | -0.27 | 0.01 | -0.29;-0.24 | 0 |
| Practice | - | O | A | -0.05 | 0.01 | -0.07;-0.03 | 0 |
| Practice | - | P | A | -0.19 | 0.01 | -0.21;-0.17 | 0 |
| Practice | - | Q | A | 0.46 | 0.01 | 0.44;0.48 | 0 |
| Practice | - | R | A | -0.07 | 0.01 | -0.09;-0.05 | 0 |
| Practice | - | S | A | 0.21 | 0.01 | 0.19;0.23 | 0 |
| Practice | - | T | A | -0.06 | 0.01 | -0.08;-0.04 | 0 |
| Practice | - | U | A | -0.15 | 0.01 | -0.17;-0.13 | 0 |
| Practice | - | V | A | -0.12 | 0.01 | -0.14;-0.10 | 0 |
| Practice | - | W | A | 0.11 | 0.01 | 0.09;0.14 | 0 |
| Practice | - | X | A | -0.04 | 0.01 | -0.06;-0.02 | 0.0001 |
| Practice | - | Y | A | -0.15 | 0.01 | -0.17;-0.13 | 0 |
| Practice | - | Z | A | -0.03 | 0.01 | -0.06;-0.00 | 0.025 |
| Practice | - | AA | A | -0.41 | 0.01 | -0.43;-0.39 | 0 |
| Practice | - | AB | A | -0.2 | 0.01 | -0.22;-0.18 | 0 |
| Practice | - | AC | A | 0.03 | 0.01 | 0.01;0.05 | 0.0125 |
| Practice | - | AD | A | -0.02 | 0.01 | -0.04;0.00 | 0.0589 |
| Practice | - | AE | A | -0.48 | 0.01 | -0.51;-0.45 | 0 |
| Practice | - | AF | A | -0.03 | 0.01 | -0.05;-0.01 | 0.0047 |
| Practice | - | AG | A | -0.05 | 0.01 | -0.07;-0.03 | 0 |
| Practice | - | AH | A | -0.03 | 0.01 | -0.05;-0.01 | 0.009 |
| Practice | - | AI | A | -0.07 | 0.01 | -0.09;-0.05 | 0 |
| Practice | - | AJ | A | -0.05 | 0.01 | -0.08;-0.03 | 0 |
| Practice | - | AK | A | 0 | 0.01 | -0.02;0.02 | 0.9028 |
| Practice | - | AL | A | -0.1 | 0.01 | -0.12;-0.08 | 0 |
| Practice | - | AM | A | -0.18 | 0.01 | -0.20;-0.16 | 0 |
| Practice | - | AN | A | 0.14 | 0.01 | 0.12;0.16 | 0 |
| Practice | - | AO | A | -0.09 | 0.01 | -0.11;-0.06 | 0 |
| Practice | - | AP | A | -0.01 | 0.01 | -0.03;0.01 | 0.4728 |
| Practice | - | AQ | A | 0.17 | 0.01 | 0.15;0.19 | 0 |
| Practice | - | AR | A | -0.09 | 0.01 | -0.11;-0.06 | 0 |
| Practice | - | AS | A | 0.14 | 0.01 | 0.12;0.16 | 0 |
| Practice | - | AT | A | 0.02 | 0.01 | 0.00;0.04 | 0.0335 |
| Practice | - | AU | A | 0.27 | 0.01 | 0.25;0.29 | 0 |
| Practice | - | AV | A | 0.13 | 0.01 | 0.10;0.15 | 0 |
| Practice | - | AW | A | -0.39 | 0.01 | -0.42;-0.37 | 0 |
| Practice | - | AX | A | 0.08 | 0.01 | 0.06;0.10 | 0 |
| Practice | - | AY | A | -0.05 | 0.01 | -0.07;-0.03 | 0 |
| Practice | - | AZ | A | -0.06 | 0.01 | -0.08;-0.04 | 0 |
| Practice | - | BA | A | -0.18 | 0.01 | -0.21;-0.16 | 0 |
| Practice | - | BB | A | 0 | 0.01 | -0.02;0.02 | 0.8668 |
| Practice | - | BC | A | -0.35 | 0.01 | -0.38;-0.33 | 0 |
| Practice | - | BD | A | -0.04 | 0.01 | -0.06;-0.02 | 0.0003 |
| Practice | - | BE | A | -0.29 | 0.01 | -0.31;-0.26 | 0 |
| Practice | - | BF | A | -0.43 | 0.01 | -0.46;-0.41 | 0 |
| Practice | - | BG | A | -0.12 | 0.01 | -0.14;-0.10 | 0 |
| Practice | - | BH | A | -0.21 | 0.01 | -0.23;-0.19 | 0 |
| Practice | - | BI | A | 0.04 | 0.01 | 0.02;0.06 | 0.0003 |
| Practice | - | BJ | A | -0.36 | 0.01 | -0.39;-0.34 | 0 |
| Practice | - | BK | A | 0.21 | 0.01 | 0.19;0.23 | 0 |
| Practice | - | BL | A | 0.45 | 0.01 | 0.43;0.47 | 0 |
| Practice | - | BM | A | 0.25 | 0.01 | 0.23;0.27 | 0 |
| Practice | - | BN | A | -0.04 | 0.01 | -0.06;-0.02 | 0.0007 |
| Practice | - | BO | A | -0.16 | 0.01 | -0.18;-0.14 | 0 |
| Practice | - | BP | A | -0.23 | 0.01 | -0.25;-0.20 | 0 |
| Practice | - | BQ | A | -0.08 | 0.01 | -0.10;-0.06 | 0 |
| Practice | - | BR | A | -0.09 | 0.01 | -0.12;-0.07 | 0 |
| Practice | - | BS | A | 0.42 | 0.01 | 0.40;0.44 | 0 |
| Practice | - | BT | A | -0.46 | 0.02 | -0.50;-0.42 | 0 |
| Practice | - | BU | A | -0.13 | 0.01 | -0.15;-0.10 | 0 |
| Practice | - | BV | A | 0.17 | 0.01 | 0.15;0.19 | 0 |
| Practice | - | BW | A | -0.17 | 0.01 | -0.19;-0.14 | 0 |
| Practice | - | BX | A | 0.15 | 0.01 | 0.13;0.17 | 0 |
| Practice | - | BY | A | 0.12 | 0.01 | 0.10;0.14 | 0 |
| Practice | - | BZ | A | -0.3 | 0.02 | -0.33;-0.27 | 0 |
| Practice | - | CA | A | -0.14 | 0.01 | -0.16;-0.12 | 0 |
| Practice | - | CB | A | 0.13 | 0.01 | 0.10;0.15 | 0 |
| Practice | - | CC | A | -0.08 | 0.01 | -0.10;-0.06 | 0 |
| Practice | - | CD | A | 0.27 | 0.01 | 0.25;0.29 | 0 |
| Practice | - | CE | A | -0.27 | 0.01 | -0.29;-0.24 | 0 |
| Practice | - | CF | A | -0.35 | 0.01 | -0.38;-0.33 | 0 |
| Practice | - | CG | A | -0.16 | 0.01 | -0.19;-0.14 | 0 |
| Practice | - | CH | A | -0.95 | 0.03 | -1.01;-0.89 | 0 |
| Practice | - | CI | A | -0.38 | 0.01 | -0.41;-0.35 | 0 |
| Practice | - | CJ | A | 0.06 | 0.01 | 0.04;0.08 | 0 |
| Practice | - | CK | A | 0.35 | 0.01 | 0.33;0.37 | 0 |
| Practice | - | CL | A | 0.16 | 0.01 | 0.14;0.18 | 0 |
| Practice | - | CM | A | -0.16 | 0.01 | -0.18;-0.14 | 0 |
| Practice | - | CN | A | -0.15 | 0.01 | -0.18;-0.13 | 0 |
| Practice | - | CO | A | 0.04 | 0.01 | 0.02;0.07 | 0.0003 |
| Practice | - | CP | A | 0.08 | 0.01 | 0.06;0.10 | 0 |
| Practice | - | CQ | A | 0.37 | 0.01 | 0.35;0.39 | 0 |
| Practice | - | CR | A | -0.01 | 0.01 | -0.04;0.01 | 0.2984 |
| Practice | - | CS | A | 0.37 | 0.01 | 0.35;0.39 | 0 |
| Practice | - | CT | A | -0.1 | 0.01 | -0.13;-0.08 | 0 |
| Practice | - | CU | A | -0.12 | 0.01 | -0.14;-0.10 | 0 |
| Practice | - | CV | A | -0.26 | 0.01 | -0.28;-0.23 | 0 |
| Practice | - | CW | A | 0.01 | 0.01 | -0.01;0.03 | 0.2252 |
| Practice | - | CX | A | 0.26 | 0.01 | 0.24;0.28 | 0 |
| Practice | - | CY | A | -0.25 | 0.01 | -0.28;-0.23 | 0 |
| Practice | - | CZ | A | 0.09 | 0.01 | 0.07;0.11 | 0 |
| Practice | - | DA | A | 0.47 | 0.01 | 0.45;0.49 | 0 |

2.2 Number of chronic diagnoses

| variable | period | level | reference | estimate | se | 95% CI | Pvalue |
| --- | --- | --- | --- | --- | --- | --- | --- |
| Intercept | 2018-2019 | - | - | -3.69 | 0.1 | -3.88;-3.50 | 0 |
| Intercept | 2020 | - | - | -3.83 | 0.1 | -4.03;-3.64 | 0 |
| Nationality | 2018-2019 | Foreign | Belgian | 0.05 | 0.02 | 0.01;0.09 | 0.0145 |
| Nationality | 2020 | Foreign | Belgian | 0.12 | 0.03 | 0.06;0.18 | 0 |
| Sex | 2018-2019 | Male | Female | 0.16 | 0.01 | 0.14;0.18 | 0 |
| Sex | 2020 | Male | Female | 0.15 | 0.02 | 0.12;0.18 | 0 |
| Age | 2018-2019 | 0-17 | 18-34 | -0.31 | 0.03 | -0.37;-0.25 | 0 |
| Age | 2018-2019 | 35-65 | 18-34 | 1.03 | 0.02 | 0.99;1.06 | 0 |
| Age | 2018-2019 | 65+ | 18-34 | 1.8 | 0.02 | 1.77;1.84 | 0 |
| Age | 2020 | 0-17 | 18-34 | -0.69 | 0.05 | -0.79;-0.58 | 0 |
| Age | 2020 | 35-65 | 18-34 | 1.07 | 0.03 | 1.01;1.12 | 0 |
| Age | 2020 | 65+ | 18-34 | 1.83 | 0.03 | 1.78;1.89 | 0 |
| Socio-economic status | 2018-2019 | Low | High | 0.36 | 0.01 | 0.34;0.39 | 0 |
| Socio-economic status | 2020 | Low | High | 0.3 | 0.02 | 0.27;0.34 | 0 |
| Center city | 2018-2019 | Yes | No | 0.05 | 0.02 | 0.01;0.08 | 0.0182 |
| Center city | 2020 | Yes | No | 0.08 | 0.02 | 0.03;0.12 | 0.0011 |
| Practice | - | B | A | -0.6 | 0.14 | -0.87;-0.33 | 0 |
| Practice | - | C | A | 0.26 | 0.1 | 0.07;0.46 | 0.0089 |
| Practice | - | D | A | -0.32 | 0.12 | -0.55;-0.09 | 0.0062 |
| Practice | - | E | A | -0.32 | 0.12 | -0.55;-0.09 | 0.0061 |
| Practice | - | F | A | 0.01 | 0.1 | -0.18;0.21 | 0.9025 |
| Practice | - | G | A | -0.14 | 0.12 | -0.37;0.08 | 0.2183 |
| Practice | - | H | A | -0.69 | 0.12 | -0.92;-0.46 | 0 |
| Practice | - | I | A | -0.11 | 0.12 | -0.35;0.14 | 0.3869 |
| Practice | - | J | A | 0.19 | 0.1 | -0.01;0.39 | 0.0667 |
| Practice | - | K | A | -0.12 | 0.1 | -0.32;0.08 | 0.2465 |
| Practice | - | L | A | -0.34 | 0.11 | -0.55;-0.12 | 0.002 |
| Practice | - | M | A | 0.19 | 0.11 | -0.02;0.39 | 0.0771 |
| Practice | - | N | A | 0.01 | 0.12 | -0.22;0.25 | 0.9214 |
| Practice | - | O | A | 0.23 | 0.1 | 0.03;0.43 | 0.0251 |
| Practice | - | P | A | 0.06 | 0.1 | -0.13;0.26 | 0.5288 |
| Practice | - | Q | A | 0.71 | 0.1 | 0.51;0.90 | 0 |
| Practice | - | R | A | 0.15 | 0.11 | -0.05;0.36 | 0.1493 |
| Practice | - | S | A | 0.35 | 0.1 | 0.15;0.55 | 0.0006 |
| Practice | - | T | A | -1.63 | 0.13 | -1.89;-1.37 | 0 |
| Practice | - | U | A | 0.39 | 0.1 | 0.20;0.59 | 0.0001 |
| Practice | - | V | A | -0.25 | 0.1 | -0.45;-0.05 | 0.0135 |
| Practice | - | W | A | -0.13 | 0.11 | -0.36;0.09 | 0.2358 |
| Practice | - | X | A | -0.23 | 0.1 | -0.43;-0.03 | 0.0233 |
| Practice | - | Y | A | 0.2 | 0.11 | -0.01;0.40 | 0.0621 |
| Practice | - | Z | A | 0.14 | 0.13 | -0.12;0.39 | 0.2862 |
| Practice | - | AA | A | 0.2 | 0.11 | -0.01;0.41 | 0.067 |
| Practice | - | AB | A | 0.12 | 0.1 | -0.08;0.33 | 0.2329 |
| Practice | - | AC | A | -0.25 | 0.1 | -0.46;-0.05 | 0.0138 |
| Practice | - | AD | A | 0.01 | 0.1 | -0.20;0.21 | 0.9591 |
| Practice | - | AE | A | -0.29 | 0.12 | -0.53;-0.05 | 0.0192 |
| Practice | - | AF | A | 0.03 | 0.1 | -0.17;0.23 | 0.781 |
| Practice | - | AG | A | 0.17 | 0.1 | -0.03;0.37 | 0.0964 |
| Practice | - | AH | A | 0.12 | 0.1 | -0.08;0.32 | 0.2297 |
| Practice | - | AI | A | -1.66 | 0.12 | -1.89;-1.42 | 0 |
| Practice | - | AJ | A | -1.01 | 0.13 | -1.28;-0.75 | 0 |
| Practice | - | AK | A | -0.17 | 0.1 | -0.38;0.03 | 0.0999 |
| Practice | - | AL | A | -0.04 | 0.1 | -0.24;0.16 | 0.7018 |
| Practice | - | AM | A | 0 | 0.11 | -0.22;0.21 | 0.9646 |
| Practice | - | AN | A | 0.1 | 0.1 | -0.09;0.30 | 0.2881 |
| Practice | - | AO | A | -0.33 | 0.11 | -0.55;-0.12 | 0.0024 |
| Practice | - | AP | A | 0.08 | 0.1 | -0.12;0.28 | 0.4265 |
| Practice | - | AQ | A | -0.01 | 0.1 | -0.21;0.18 | 0.8992 |
| Practice | - | AR | A | 0.06 | 0.11 | -0.15;0.28 | 0.5817 |
| Practice | - | AS | A | 0.19 | 0.11 | -0.02;0.40 | 0.0771 |
| Practice | - | AT | A | -0.03 | 0.1 | -0.23;0.17 | 0.7847 |
| Practice | - | AU | A | 0.25 | 0.11 | 0.04;0.46 | 0.0194 |
| Practice | - | AV | A | -0.25 | 0.12 | -0.48;-0.01 | 0.0396 |
| Practice | - | AW | A | 0.59 | 0.11 | 0.38;0.80 | 0 |
| Practice | - | AX | A | 0.7 | 0.1 | 0.50;0.90 | 0 |
| Practice | - | AY | A | 0.34 | 0.11 | 0.13;0.56 | 0.0015 |
| Practice | - | AZ | A | 0.11 | 0.11 | -0.10;0.31 | 0.3169 |
| Practice | - | BA | A | 0.03 | 0.11 | -0.19;0.26 | 0.7578 |
| Practice | - | BB | A | -0.46 | 0.12 | -0.70;-0.22 | 0.0001 |
| Practice | - | BC | A | -0.57 | 0.14 | -0.84;-0.31 | 0 |
| Practice | - | BD | A | 0.07 | 0.11 | -0.13;0.28 | 0.4882 |
| Practice | - | BE | A | -0.15 | 0.11 | -0.38;0.08 | 0.1911 |
| Practice | - | BF | A | -0.96 | 0.13 | -1.22;-0.71 | 0 |
| Practice | - | BG | A | 0.1 | 0.1 | -0.09;0.30 | 0.3026 |
| Practice | - | BH | A | 0.02 | 0.1 | -0.18;0.22 | 0.85 |
| Practice | - | BI | A | -0.37 | 0.12 | -0.60;-0.15 | 0.0013 |
| Practice | - | BJ | A | -0.72 | 0.11 | -0.93;-0.51 | 0 |
| Practice | - | BK | A | 0.07 | 0.1 | -0.13;0.27 | 0.4974 |
| Practice | - | BL | A | 0.35 | 0.11 | 0.14;0.56 | 0.0012 |
| Practice | - | BM | A | 0.09 | 0.1 | -0.11;0.29 | 0.3604 |
| Practice | - | BN | A | -0.05 | 0.11 | -0.25;0.16 | 0.6573 |
| Practice | - | BO | A | -0.19 | 0.11 | -0.40;0.02 | 0.0768 |
| Practice | - | BP | A | -0.13 | 0.12 | -0.38;0.11 | 0.2781 |
| Practice | - | BQ | A | -0.74 | 0.11 | -0.95;-0.53 | 0 |
| Practice | - | BR | A | -0.45 | 0.11 | -0.66;-0.24 | 0 |
| Practice | - | BS | A | 0.14 | 0.11 | -0.08;0.36 | 0.1999 |
| Practice | - | BT | A | 0.22 | 0.16 | -0.09;0.54 | 0.1621 |
| Practice | - | BU | A | -1.29 | 0.16 | -1.60;-0.98 | 0 |
| Practice | - | BV | A | 0.98 | 0.1 | 0.78;1.19 | 0 |
| Practice | - | BW | A | -0.09 | 0.11 | -0.31;0.13 | 0.4214 |
| Practice | - | BX | A | -0.46 | 0.11 | -0.67;-0.25 | 0 |
| Practice | - | BY | A | 0.57 | 0.1 | 0.36;0.78 | 0 |
| Practice | - | BZ | A | -0.71 | 0.16 | -1.03;-0.39 | 0 |
| Practice | - | CA | A | -0.24 | 0.1 | -0.44;-0.04 | 0.0176 |
| Practice | - | CB | A | 0.48 | 0.11 | 0.27;0.68 | 0 |
| Practice | - | CC | A | 0.11 | 0.11 | -0.10;0.32 | 0.2961 |
| Practice | - | CD | A | 0.24 | 0.1 | 0.04;0.44 | 0.0178 |
| Practice | - | CE | A | -0.12 | 0.11 | -0.34;0.09 | 0.2682 |
| Practice | - | CF | A | -0.44 | 0.13 | -0.69;-0.19 | 0.0005 |
| Practice | - | CG | A | -0.29 | 0.11 | -0.51;-0.08 | 0.0079 |
| Practice | - | CH | A | -1.42 | 0.37 | -2.14;-0.71 | 0.0001 |
| Practice | - | CI | A | -0.1 | 0.12 | -0.34;0.14 | 0.4146 |
| Practice | - | CJ | A | -0.08 | 0.11 | -0.29;0.13 | 0.4483 |
| Practice | - | CK | A | 0.5 | 0.1 | 0.30;0.70 | 0 |
| Practice | - | CL | A | 0.17 | 0.11 | -0.04;0.38 | 0.1111 |
| Practice | - | CM | A | -0.83 | 0.11 | -1.05;-0.61 | 0 |
| Practice | - | CN | A | -3.90E-01 | 0.13 | -0.66;-0.13 | 0.0033 |
| Practice | - | CO | A | -8.00E-02 | 0.12 | -0.31;0.15 | 0.5089 |
| Practice | - | CP | A | 2.60E-01 | 0.1 | 0.07;0.45 | 0.0084 |
| Practice | - | CQ | A | 0.47 | 0.11 | 0.27;0.68 | 0 |
| Practice | - | CR | A | -0.7 | 0.13 | -0.95;-0.44 | 0 |
| Practice | - | CS | A | 3.50E-01 | 0.11 | 0.13;0.57 | 0.002 |
| Practice | - | CT | A | -0.93 | 0.15 | -1.21;-0.64 | 0 |
| Practice | - | CU | A | -1.21 | 0.13 | -1.46;-0.97 | 0 |
| Practice | - | CV | A | -0.24 | 0.11 | -0.45;-0.03 | 0.0232 |
| Practice | - | CW | A | 2.70E-01 | 0.1 | 0.07;0.46 | 0.0089 |
| Practice | - | CX | A | 0.12 | 0.11 | -0.10;0.33 | 0.2787 |
| Practice | - | CY | A | -1.01 | 0.14 | -1.29;-0.73 | 0 |
| Practice | - | CZ | A | 0.15 | 0.1 | -0.06;0.35 | 0.1548 |
| Practice | - | DA | A | 0.3 | 0.1 | 0.10;0.50 | 0.0037 |

2.3 Number of acute diagnoses

| variable | period | level | reference | estimate | se | 95% CI | Pvalue |
| --- | --- | --- | --- | --- | --- | --- | --- |
| Intercept | 2018-2019 | - | - | 0.7 | 0.02 | 0.67;0.74 | 0 |
| Intercept | 2020 | - | - | 0.75 | 0.02 | 0.72;0.79 | 0 |
| Nationality | 2018-2019 | Foreign | Belgian | -0.06 | 0 | -0.07;-0.05 | 0 |
| Nationality | 2020 | Foreign | Belgian | -0.02 | 0.01 | -0.03;-0.01 | 0.0002 |
| Sex | 2018-2019 | Male | Female | -0.14 | 0 | -0.15;-0.14 | 0 |
| Sex | 2020 | Male | Female | -0.16 | 0 | -0.17;-0.16 | 0 |
| Age | 2018-2019 | 0-17 | 18-34 | -0.03 | 0 | -0.04;-0.02 | 0 |
| Age | 2018-2019 | 35-65 | 18-34 | -0.03 | 0 | -0.03;-0.02 | 0 |
| Age | 2018-2019 | 65+ | 18-34 | -0.07 | 0 | -0.07;-0.06 | 0 |
| Age | 2020 | 0-17 | 18-34 | -0.15 | 0.01 | -0.16;-0.14 | 0 |
| Age | 2020 | 35-65 | 18-34 | -0.02 | 0 | -0.03;-0.01 | 0 |
| Age | 2020 | 65+ | 18-34 | -0.09 | 0 | -0.10;-0.08 | 0 |
| Socio-economic status | 2018-2019 | Low | High | 0.19 | 0 | 0.18;0.20 | 0 |
| Socio-economic status | 2020 | Low | High | 0.19 | 0 | 0.18;0.20 | 0 |
| Center city | 2018-2019 | Yes | No | 0 | 0 | -0.01;0.00 | 0.4052 |
| Center city | 2020 | Yes | No | 0.04 | 0 | 0.03;0.05 | 0 |
| Practice | - | B | A | -0.18 | 0.02 | -0.23;-0.13 | 0 |
| Practice | - | C | A | 0.1 | 0.02 | 0.06;0.14 | 0 |
| Practice | - | D | A | -1.24 | 0.03 | -1.30;-1.18 | 0 |
| Practice | - | E | A | -1.41 | 0.03 | -1.47;-1.34 | 0 |
| Practice | - | F | A | -0.22 | 0.02 | -0.26;-0.18 | 0 |
| Practice | - | G | A | -0.69 | 0.02 | -0.74;-0.64 | 0 |
| Practice | - | H | A | -1.12 | 0.03 | -1.17;-1.07 | 0 |
| Practice | - | I | A | -0.48 | 0.03 | -0.54;-0.43 | 0 |
| Practice | - | J | A | 0.1 | 0.02 | 0.06;0.14 | 0 |
| Practice | - | K | A | 0.14 | 0.02 | 0.10;0.17 | 0 |
| Practice | - | L | A | -0.08 | 0.02 | -0.12;-0.04 | 0.0001 |
| Practice | - | M | A | -1.12 | 0.03 | -1.17;-1.07 | 0 |
| Practice | - | N | A | -0.22 | 0.03 | -0.27;-0.17 | 0 |
| Practice | - | O | A | 0.19 | 0.02 | 0.15;0.23 | 0 |
| Practice | - | P | A | 0.02 | 0.02 | -0.02;0.05 | 0.4193 |
| Practice | - | Q | A | 0.72 | 0.02 | 0.68;0.75 | 0 |
| Practice | - | R | A | -0.38 | 0.02 | -0.43;-0.34 | 0 |
| Practice | - | S | A | 0.59 | 0.02 | 0.56;0.63 | 0 |
| Practice | - | T | A | -1.69 | 0.03 | -1.74;-1.64 | 0 |
| Practice | - | U | A | 0.02 | 0.02 | -0.02;0.06 | 0.3498 |
| Practice | - | V | A | -0.33 | 0.02 | -0.37;-0.29 | 0 |
| Practice | - | W | A | -0.48 | 0.02 | -0.52;-0.43 | 0 |
| Practice | - | X | A | -0.62 | 0.02 | -0.66;-0.59 | 0 |
| Practice | - | Y | A | -0.14 | 0.02 | -0.18;-0.10 | 0 |
| Practice | - | Z | A | 0.26 | 0.02 | 0.22;0.31 | 0 |
| Practice | - | AA | A | -0.16 | 0.02 | -0.20;-0.12 | 0 |
| Practice | - | AB | A | 0.37 | 0.02 | 0.33;0.41 | 0 |
| Practice | - | AC | A | -0.69 | 0.02 | -0.73;-0.65 | 0 |
| Practice | - | AD | A | -0.33 | 0.02 | -0.37;-0.29 | 0 |
| Practice | - | AE | A | -0.55 | 0.03 | -0.60;-0.49 | 0 |
| Practice | - | AF | A | -0.47 | 0.02 | -0.51;-0.43 | 0 |
| Practice | - | AG | A | 0.07 | 0.02 | 0.03;0.11 | 0.0003 |
| Practice | - | AH | A | -0.22 | 0.02 | -0.26;-0.18 | 0 |
| Practice | - | AI | A | -1.4 | 0.02 | -1.44;-1.36 | 0 |
| Practice | - | AJ | A | -1.52 | 0.03 | -1.58;-1.46 | 0 |
| Practice | - | AK | A | -0.44 | 0.02 | -0.48;-0.40 | 0 |
| Practice | - | AL | A | -0.13 | 0.02 | -0.17;-0.09 | 0 |
| Practice | - | AM | A | 0.32 | 0.02 | 0.28;0.36 | 0 |
| Practice | - | AN | A | 0.05 | 0.02 | 0.02;0.09 | 0.0057 |
| Practice | - | AO | A | -1.2 | 0.03 | -1.25;-1.15 | 0 |
| Practice | - | AP | A | -0.72 | 0.02 | -0.77;-0.68 | 0 |
| Practice | - | AQ | A | 0.57 | 0.02 | 0.53;0.61 | 0 |
| Practice | - | AR | A | -0.06 | 0.02 | -0.11;-0.02 | 0.0048 |
| Practice | - | AS | A | 0.61 | 0.02 | 0.57;0.65 | 0 |
| Practice | - | AT | A | 0.28 | 0.02 | 0.24;0.31 | 0 |
| Practice | - | AU | A | 0.34 | 0.02 | 0.31;0.38 | 0 |
| Practice | - | AV | A | -0.01 | 0.02 | -0.06;0.03 | 0.5728 |
| Practice | - | AW | A | -0.1 | 0.02 | -0.15;-0.06 | 0 |
| Practice | - | AX | A | 0.21 | 0.02 | 0.17;0.25 | 0 |
| Practice | - | AY | A | 0.5 | 0.02 | 0.46;0.54 | 0 |
| Practice | - | AZ | A | 0.03 | 0.02 | -0.01;0.07 | 0.0979 |
| Practice | - | BA | A | -0.82 | 0.03 | -0.87;-0.76 | 0 |
| Practice | - | BB | A | -0.65 | 0.02 | -0.70;-0.60 | 0 |
| Practice | - | BC | A | -0.65 | 0.03 | -0.71;-0.60 | 0 |
| Practice | - | BD | A | -0.1 | 0.02 | -0.14;-0.06 | 0 |
| Practice | - | BE | A | -0.05 | 0.02 | -0.09;-0.00 | 0.0446 |
| Practice | - | BF | A | -1.57 | 0.03 | -1.63;-1.51 | 0 |
| Practice | - | BG | A | -0.23 | 0.02 | -0.27;-0.19 | 0 |
| Practice | - | BH | A | -0.3 | 0.02 | -0.34;-0.26 | 0 |
| Practice | - | BI | A | -0.03 | 0.02 | -0.07;0.01 | 0.1362 |
| Practice | - | BJ | A | -0.92 | 0.02 | -0.97;-0.88 | 0 |
| Practice | - | BK | A | 0.14 | 0.02 | 0.10;0.18 | 0 |
| Practice | - | BL | A | 0.09 | 0.02 | 0.05;0.14 | 0 |
| Practice | - | BM | A | 0.3 | 0.02 | 0.26;0.34 | 0 |
| Practice | - | BN | A | -0.4 | 0.02 | -0.45;-0.36 | 0 |
| Practice | - | BO | A | -0.35 | 0.02 | -0.39;-0.30 | 0 |
| Practice | - | BP | A | -0.73 | 0.03 | -0.79;-0.67 | 0 |
| Practice | - | BQ | A | -0.65 | 0.02 | -0.69;-0.61 | 0 |
| Practice | - | BR | A | -0.71 | 0.02 | -0.75;-0.67 | 0 |
| Practice | - | BS | A | 0.22 | 0.02 | 0.18;0.27 | 0 |
| Practice | - | BT | A | -0.69 | 0.04 | -0.77;-0.61 | 0 |
| Practice | - | BU | A | -0.57 | 0.03 | -0.62;-0.51 | 0 |
| Practice | - | BV | A | 0.39 | 0.02 | 0.35;0.43 | 0 |
| Practice | - | BW | A | -0.26 | 0.02 | -0.31;-0.22 | 0 |
| Practice | - | BX | A | -0.3 | 0.02 | -0.34;-0.26 | 0 |
| Practice | - | BY | A | 0.49 | 0.02 | 0.45;0.53 | 0 |
| Practice | - | BZ | A | -0.65 | 0.03 | -0.71;-0.58 | 0 |
| Practice | - | CA | A | -0.23 | 0.02 | -0.27;-0.19 | 0 |
| Practice | - | CB | A | 0.22 | 0.02 | 0.18;0.26 | 0 |
| Practice | - | CC | A | -0.15 | 0.02 | -0.19;-0.11 | 0 |
| Practice | - | CD | A | 0.22 | 0.02 | 0.18;0.26 | 0 |
| Practice | - | CE | A | -0.31 | 0.02 | -0.36;-0.27 | 0 |
| Practice | - | CF | A | -0.38 | 0.02 | -0.43;-0.33 | 0 |
| Practice | - | CG | A | -0.39 | 0.02 | -0.44;-0.35 | 0 |
| Practice | - | CH | A | -1.05 | 0.06 | -1.16;-0.93 | 0 |
| Practice | - | CI | A | 0.41 | 0.02 | 0.36;0.45 | 0 |
| Practice | - | CJ | A | -0.53 | 0.02 | -0.57;-0.49 | 0 |
| Practice | - | CK | A | 0.27 | 0.02 | 0.23;0.31 | 0 |
| Practice | - | CL | A | -0.16 | 0.02 | -0.20;-0.12 | 0 |
| Practice | - | CM | A | -0.88 | 0.02 | -0.93;-0.84 | 0 |
| Practice | - | CN | A | -0.51 | 0.03 | -0.56;-0.45 | 0 |
| Practice | - | CO | A | 0.05 | 0.02 | 0.01;0.10 | 0.0213 |
| Practice | - | CP | A | 0.38 | 0.02 | 0.34;0.42 | 0 |
| Practice | - | CQ | A | 0.45 | 0.02 | 0.41;0.49 | 0 |
| Practice | - | CR | A | -0.86 | 0.03 | -0.91;-0.81 | 0 |
| Practice | - | CS | A | 0.46 | 0.02 | 0.42;0.50 | 0 |
| Practice | - | CT | A | -0.51 | 0.03 | -0.56;-0.46 | 0 |
| Practice | - | CU | A | -1.06 | 0.02 | -1.11;-1.01 | 0 |
| Practice | - | CV | A | -0.84 | 0.02 | -0.89;-0.80 | 0 |
| Practice | - | CW | A | 0.15 | 0.02 | 0.11;0.19 | 0 |
| Practice | - | CX | A | -0.02 | 0.02 | -0.06;0.03 | 0.4388 |
| Practice | - | CY | A | -0.23 | 0.02 | -0.28;-0.19 | 0 |
| Practice | - | CZ | A | 0.07 | 0.02 | 0.03;0.11 | 0.0008 |
| Practice | - | DA | A | 0.08 | 0.02 | 0.04;0.12 | 0.0001 |
